# Contribution of Positron Emission Tomography-Computed Tomography (PET/CT) to the Diagnosis of Endocarditis Due to an Infected Pacemaker Associated With Spondylodiscitis

**DOI:** 10.7759/cureus.43140

**Published:** 2023-08-08

**Authors:** Antonios Kalkinis, George Vassilopoulos, Stelios Rokas, Christos Koutserimpas, George Samonis

**Affiliations:** 1 Department of Nuclear Medicine, Metropolitan Hospital, Athens, GRC; 2 Department of Cardiology, Metropolitan Hospital, Athens, GRC; 3 Department of Orthopaedics and Traumatology, 251 Hellenic Air Force General Hospital, Athens, GRC; 4 Department of Oncology, Metropolitan Hospital, Athens, GRC

**Keywords:** nuclear medicine imaging, pet/ct, electronic devices, cardiac implantation, endocarditis

## Abstract

The incidence of cardiac device-related endocarditis (CDRIE) is increasing, and its diagnosis and treatment may occasionally be problematic. Echocardiography is important for its diagnosis, and 18F-fluorodeoxyglucose positron emission tomography-computed tomography (PET/CT) may also be useful as a diagnostic procedure. A case of CDRIE due to an infected pacemaker is presented. In this case, blood cultures were repeatedly negative, and transesophageal ultrasound examination did not reveal signs of the disease. However, PET/CT revealed the infection. The causative organism was *Corynebacterium* spp, and this was finally identified by polymerase chain reaction of a sample of the device material. Eight weeks before the development of CDRIE symptoms, the patient had been empirically treated for spondylodiscitis caused by a non-identified organism. CDRIE and spondylodiscitis are closely associated infections. The present case of CDRIE was treated successfully with six weeks of combination antimicrobial treatment. PET/CT may contribute to CDRIE diagnosis by locating the site(s) of the infection, especially in cases that are ultrasound and culture negative.

## Introduction

Infective endocarditis (IE) is an infection involving the endocardial surface of the heart. Manifestations may affect several organ systems, including malaise, myalgia, arthralgia, anorexia, night sweats, and headaches, whereas those of the heart include valvular vegetation, murmurs, abscesses, periannular infection, and myopericarditis [1]. The causative organisms are usually streptococci, staphylococci, and enterococci, whereas in recent years, the predominance of streptococcal infections, often associated with invasive procedures, was reported [2,3]. IE may present as an acute, rapidly progressive, infection or as subacute or chronic disease, with low-grade fever and nonspecific symptoms. Most patients present with fever, often accompanied by systemic symptoms, such as poor appetite and weight loss. IE may be associated with significant morbidity and mortality (short-term mortality ranging between 10 and 30%) [2-5]. With an aging population and increasing use of implantable devices and heart valves, the incidence of this infection is increasing [4,5].

Cardiac implantable electronic devices, such as pacemakers, cardioverter-defibrillators, and cardiac resynchronization therapy devices, represent significant progress because they can manage life-threatening bradycardia. However, their use can be related to complications, including the infection of some or all of the parts of the device, resulting in IE [6]. As the use of such items has increased, so has the incidence of cardiac device-related endocarditis (CDRIE), ranging between 0.1% and 5.1%, the diagnosis and treatment of which can be problematic [6]. Early suspicion and rapid diagnosis are critical to enable prompt treatment, including causative antimicrobial therapy, removal of any infected implanted devices, and monitoring response to antibiotic therapy and of the hemodynamic and cardiac status, which reduces complications and mortality [3-6].

Imaging modalities, such as echocardiography, are important for the diagnosis; however, 18F-fluorodeoxyglucose positron emission tomography-computed tomography (18F-FDG PET/CT) represents a useful diagnostic tool [1].

Imaging technics may be helpful, although positive blood cultures represent the cornerstone of diagnosis. Culture-negative IE (12% to 20%) or CDRIE, although relatively rare, may cause diagnostic and therapeutic problems.

The aim of the present case report was to raise the awareness of CDRIE among clinicians and to provide appropriate information regarding the diagnosis and management of this severe infection.

## Case presentation

A 69-year-old Caucasian female had a pacemaker implanted 11 years ago, due to complete heart block. However, one month later, the device had a malfunction, and the lead was reinstalled. Eighteen months later, signs of pocket erosion were noticed, and the generator was successfully reimplanted under the pectoralis muscle in the ipsilateral side. Ten years later, she complained of intense back pain and magnetic resonance imaging of the spine revealed spondylodiscitis of the 11th and 12th thoracic vertebrae. Blood cultures, as well as those from the infected spinal tissues, during an antimicrobial-free period, did not reveal the causative microorganism(s). Therefore, the infection was empirically treated with intravenous (IV) daptomycin and meropenem, for 40 days, with a successful clinical outcome.

**Figure 1:**
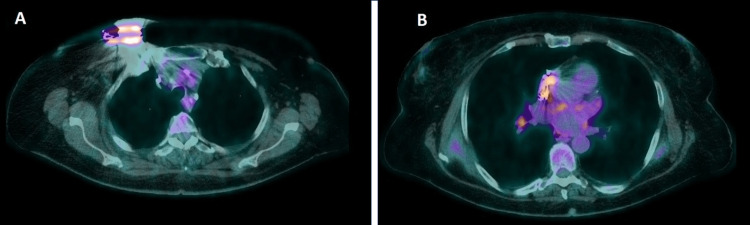
Transaxial views of fused 18F-FDG PET/CT images revealing signs suggestive of endocarditis due to an infected pacemaker. Findings (A and B) strongly support the diagnosis of CDRIE in the current clinical context. 18F-FDG PET/CT: Fluorine 18-fluorodeoxyglucose positron emission tomography/computed tomography. CDRIE:  Culture-negative endocarditis due to the infected pacemaker.

At that point, depletion of the pacemaker’s generator was detected, and a new (third) adjustment was made on the same side, eight weeks after the spondylodiscitis onset, after ensuring resolution of the infection. Seven days after the pacemaker adjustment, signs of pocket infection were observed, including skin erythema, pain, edema, and purulent drainage in the pocket area, without symptoms of systemic infection (blood pressure: 135/78 mmHg, heart rate= 80 beats per min). The patient did not have anemia, and the white blood cell count, c-reactive protein (CRP), and erythrocyte sedimentation rate (ESR) were within normal limits, while blood and drainage cultures were repeatedly negative. Transesophageal echocardiography did not reveal any signs of endocarditis. In order to confirm and document the suspected diagnosis of IE and to exclude other possible sites of infection, PET/CT was performed, revealing an increased uptake from the pacemaker generator and the electrodes inside the right atrium and the right ventricle, which strongly supported a diagnosis of CDRIE (Figure 1). Therefore, the pacemaker was removed (percutaneous extraction) and a temporary one was fitted to the femoral vein to support the cardiac function. Cultures of all parts of the device were negative and empirical treatment with IV meropenem and daptomycin combination was initiated. However, 24 hours later, polymerase chain reaction (PCR) examination of several parts of the device revealed Corynebacterium spp. Because the strain was not available for sensitivity testing, and taking into account the possible resistance of Corynebacterium to daptomycin, this agent was replaced with vancomycin. The treatment was continued for 40 days with a successful outcome (no signs or symptoms of infection). No signs or symptoms of embolic manifestation were present during the treatment period. 

Following completion of treatment, a new pacemaker system was implanted on the contralateral side of the thorax. Three years later, she is in excellent clinical condition (without any signs or symptoms of IE or any other disease), without any problems from the pacemaker which is annually checked.

## Discussion

Here, we present a case of culture-negative CDRIE, revealed by PET/CT and confirmed by PCR examination of the material from an infected device, which identified Corynebacterium spp as the causative organism. The species was not characterized, since general PCR, specific only to the genus, was used.

Blood culture-negative endocarditis may account for 2.5% to 70% of all IE cases (mainly reported between 12 and 20%), depending on reports from different geographical areas [7,8].

Blood cultures yielding Corynebacterium spp are often considered to be a result of contamination because this organism is omnipresent in the skin flora. However, severe infections due to Corynebacterium spp, such as IE and sepsis, were reported recently, mainly due to the increasing incidence of invasive techniques [9,10]. In particular, a limited number of CDRIE due to Corynebacterium spp have been reported. Most of these cases were treated with vancomycin and all of them with device removal within the first inpatient days. 

Corynebacterium species are aerobic, non-sporulating, pleomorphic, Gram-positive bacilli that are considered components of normal skin flora. Recognized risk factors for Corynebacterium endocarditis include preexisting cardiac disease, a history of bacterial endocarditis, and the presence of prosthetic devices. However, it is becoming an emerging infectious agent, a multidrug-resistant pathogen with the capacity to form biofilms, since it has been reported to be resistant to several antimicrobials, including beta-lactams, quinolones, daptomycin, gentamicin, and rifampicin [9,11]. Vancomycin is usually active in vitro, whereas Corynebacterium spp resistance to this agent is extremely rare [9].

The present patient was treated empirically with a combination of daptomycin and meropenem, which theoretically, is an efficient regimen. Daptomycin is bactericidal for most Gram-positive organisms [12], whereas meropenem has broad activity against most Gram-negative bacteria [13]. However, because the sensitivities of the organism remained unknown and the resistance of Corynebacterium spp to daptomycin was reported, this agent was replaced with vancomycin, which is the agent of choice for this infection [12]. The patient received the regimen for six weeks [14].

Spondylodiscitis is an infection with increasing incidence (0.4 to 2.4/100,000 people), due to longer life expectancy, which is associated with increased multimorbidity [15]. IE following or coexisting with spondylodiscitis is not unusual. It was reported that IE may be present in up to 30% of spondylodiscitis cases [16]. In the present case, the causative organism of spondylodiscitis was not identified. However, empirical treatment for an adequate period led to a clinically favorable outcome. The vertebral infection preceded the CDRIE. The rather short interval between the two infections could suggest that there could be a connection. The pathogenesis connecting the two infections remains unclear and a matter of debate. However, irrespective of the exact pathogenesis, the association of spondylodiscitis and IE should always be considered, especially in cases with suspicious relevant symptoms [15, 17-19]. Nevertheless, in the present case, the multiple adjustments of the pacemaker on the same side cannot preclude the device infection from existing skin flora. It has been reported that a six-week antimicrobial treatment of spondylodiscitis-associated IE has been efficient without the increased risk of relapse [17-19]. Hence, the present patient was treated with an appropriate antimicrobial combination for 40 days, followed by the installation of a new pacemaker on the contralateral side,

Regarding CDRIE, most studies recommend device and lead replacement for radical treatment as in the present case [20]. The criteria for CDRIE diagnosis are not uniform in different studies. However, most researchers agree that the four most important points that should exist to diagnose CDRIE are the presence of a cardiac device, no other obvious site of infection, cultures yielding the causative organism(s) from blood, the pocket, or the leads of the device and, finally, echocardiographic findings suggesting IE [20]. In particular, according to the 2019 International cardiac implantable electronic device infection criteria, generator pocket swelling, erythema, warmth, pain, and purulent discharge/sinus formation or deformation of pocket, adherence, and threatened erosion or exposed generator or proximal leads as diagnostic of a definite CDRIE. Furthermore, intracardiac echocardiography can be utilized for detecting vegetations, while PET/CT can identify abnormal activity in the pocket or along leads that would be suggestive of CDRIE [21].

The signs and symptoms of CDRIE are usually different from those of typical IE. The most common sign is fever with a long duration. Many patients, when symptoms first appear, are investigated for fever of unknown origin. Often, tenderness and/or symptoms of inflammation of the tissues around the device entrance to the body may be present, due to local infection. Splenomegaly and signs of peripheral emboli are rare. Elevated ESR is uncommon, although leukocytosis, anemia, and microscopic hematuria may be observed; however, they are less common than in typical valvular IE. Pulmonary emboli, often asymptomatic, may be present, either in the first instance or iatrogenically after the removal of the lead [6]. The present case had obvious signs of local infection of the pacemaker; however, she did not develop fever or other typical symptoms of CDRIE.

Treatment includes the administration of appropriate antimicrobial agents, usually in combination with removal of the electrical leads, with or without the whole device [6]. In the present case, the whole device was removed in combination with antimicrobial treatment.

Transthoracic echocardiography (TTE), especially transesophageal echocardiography (TEE), is the diagnostic procedure of choice. It can reveal vegetations, abscesses, or pseudoaneurysms. However, less accurate diagnostic findings of TTE and TEE have been reported in cases of infected prosthetic valve or implantable cardiac electronic devices where findings may be normal or inconclusive [4], as in the present case.

PET-CT has had an increasing role in the identification of several infections, including CDRIE [20]. PET/CT is probably insufficient for the diagnosis of IE, but it can be useful for the diagnosis of PV endocarditis and skin or ICED pocket infections [20,22]. PET/CT as a diagnostic tool for CDRIE had 87% sensitivity and 94% specificity in a recent meta-analysis [21]. PET/CT has better diagnostic accuracy for pocket infections than those of lead. In particular, the sensitivity and specificity for pocket infections were 93% and 98%, respectively, whereas sensitivity and specificity for the lead were 65% and 88%, respectively. It is of note that regarding contraindications of PET/CT only pregnancy is considered an absolute one, while others such as obesity may reduce image quality and interpretation [22]. Therefore, PET/CT may contribute significantly to the diagnosis of CDRIE as in the present case. However, it should be mentioned that, although PET/CT contributed to diagnosis, clinical presentation is of utmost importance in the decision-making process.

Finally, it must be noted that, despite all advances in diagnostic and therapeutic procedures, IE and CDRIE remain severe life-threatening diseases and their outcome has marginally improved during the last decade [20].

## Conclusions

The diagnosis of CDRIE occasionally may be problematic. The signs and symptoms of CDRIE may vary from those of typical IE, with the most common sign being a long-lasting fever. PET/CT may contribute to its diagnosis, by locating the site(s) of infection, including the generator pocket, especially in echocardiography and culture-negative cases. Medical therapy alone is associated with a high risk of recurrence. Complete removal of the system in combination with antimicrobial agents is the treatment of choice.
